# Knowledge on Pre-Hospital Emergency Management of Tooth Avulsion among Croatian Students of the Faculty of Education

**DOI:** 10.3390/ijerph17197159

**Published:** 2020-09-30

**Authors:** Zvonimir Uzarevic, Zrinka Ivanisevic, Matej Karl, Marina Tukara, Dora Karl, Marko Matijevic

**Affiliations:** 1 Faculty of Education, University of Osijek, Cara Hadrijana 10, 31000 Osijek, Croatia; mtukara@foozos.hr; 2 Faculty of Dental Medicine and Health, University of Osijek, 31000 Osijek, Croatia; zrinkaivan@gmail.com (Z.I.); karlmatej@gmail.com (M.K.); marko.matijevic@fdmz.hr (M.M.); 3 Faculty of Medicine Osijek, University of Osijek, 31000 Osijek, Croatia; dora734@gmail.com

**Keywords:** knocked-out tooth, tooth injuries, dental trauma, questionnaire

## Abstract

The purpose of this study was to evaluate the knowledge regarding tooth avulsion and dental first aid response among Croatian students of the Faculty of Education. A cross-sectional study was conducted among students. Participants (N = 235) were female with an average age of 21.9 ± 2.7 years. The questionnaire contained 10 close-ended questions with two to eleven possible answers. Every participant chose one correct answer. Statistical significance was determined using a Chi-square test. Majority of participants had never received any kind of information on management of a knocked-out tooth. Questions in the nature of tooth injuries, a knocked-out tooth, tooth replantation and whether the knocked-out tooth should be placed back were answered confirmatively by 40.43%, 83.40%, 57.02% and 62.55% of participants, respectively. The questionnaire showed that 18.78% of participants were aware that replantation should be performed within 30 min. The appropriate cleaning and transport medium was chosen by 40.85% and 35.31% of participants, respectively. A nearby dentist would be visited by 66.38% of students. Only 8.08% of participants provided a correct answer to all of the knowledge-based questions. The “I do not know” answer was chosen by 18.30% of participants whenever it was offered. This indicates that the majority (89.78%) were not aware of the fact that the procedure they chose would be inappropriate. The current study confirmed that future primary school teachers have a lack of knowledge for immediate response to tooth avulsion, leaving small chances for a successful prognosis of tooth replantation.

## 1. Introduction

Oral trauma accounts for around 5% of all bodily injuries, with even higher occurrence among young children [1]. Traumatic tooth injuries comprise 95% of all oral injuries and have a high incidence [1,2]. Traumatic tooth injuries are usually not a life-threatening emergency, and for that reason, it may be perceived as a less urgent condition by the public, as well as the staff in emergency departments [3]. However, delay in providing appropriate care has been found to significantly jeopardize the treatment outcome and cause more complications [4,5,6,7,8]. This will have negative consequences not only for the tooth but also on the growth and development of the alveolar bone, which would accordingly affect future treatment choices and outcomes and cause functional and esthetic problems [9,10,11,12].

Knowing how to manage tooth avulsion accurately and emergently is important among teachers, coaches, physicians as well as parents and other non-professionals, but it is especially vital for students of education as future childcare professionals. Research shows that 48.45% of traumatic dental injuries occur during childhood before the age of ten. [13]. Among dental injuries, 4 to 22% of tooth avulsions were recorded in various studies [14]. Students of the Faculty of Education are being educated to teach pupils from 1st to 4th grade. Children start attending school at age six, which means that students who will become future teachers will probably encounter permanent tooth avulsion.

The International Association of Dental Traumatology (IADT) has published guidelines for the management of permanent teeth avulsion [15]. Following their instructions, an avulsed tooth should be found immediately, washed out briefly if it is dirty and either replanted at the site or put in a cup with milk or another suitable medium for tooth transport, prior to visiting a dentist. Frequent low awareness of the proper response to tooth avulsion is reported among different populations of non-professional participants [16,17,18,19,20,21,22]. Immediate response by a person witnessing the accident can play an important role in the possibility of successful tooth replantation.

The aim of this study is to evaluate via a questionnaire the knowledge regarding tooth avulsion and emergency response among Croatian students from the Faculty of Education.

## 2. Participants and Methods

### 2.1. Participants

A cross-sectional study was conducted among Croatian students from the Faculty of Education. Students were eligible to participate and informed by personal email about the online questionnaire. All participants (N = 235) were female with average age of 21.9 ± 2.7 years. Students were asked to fill out the self-administered questionnaire.

### 2.2. Questionnaire Procedures

The objectives of the study and information on its purpose were explained to students at the beginning of the questionnaire. The voluntary nature of the study was emphasized, and confidentiality was assured. The participating students could withdraw from the study at any time. All participants were over 18 years old. The questionnaire was adopted [16,17,18] and with minor changes it was translated into the local language, Croatian. Participants answered 10 close-ended questions with 2 to 11 possible answers. The first part assessed students’ general experience with dental injuries (yes/no questions). The second part contained knowledge-based questions and included four questions with multiple correct answers.

### 2.3. Data Collection and Statistical Analysis

Every participant chose only one correct answer. The answers were counted and a percentage for each question was calculated. A Chi-square test was used to determine whether there is a statistically significant difference between the expected and the observed frequencies of incorrect and correct responses for each knowledge-based question. Answers “I do not know” were not included. Dependency between dependent variables (knowledge-based questions) and explanatory variables (yes/no questions) was assessed using a Chi-square with Yates’ correction. Statistical analysis was performed using the Statistical Package for the Social Sciences software (version 17, SPSS Inc., Chicago, IL, USA). The level of significance was set to *p* < 0.05.

## 3. Results

The research was conducted with 235 students. More than 50% of participants confirmed that they knew what a knocked-out tooth was, what tooth replantation was and that the knocked-out tooth should be placed back into the socket (Table 1). Less than 50% of participants knew what tooth injuries were and what needed to be done if a knocked-out tooth falls on the ground. Only 22.13% of participants answered that they did receive some kind of information on management of a knocked-out tooth.

Regarding the questions about the optimum time within which a knocked-out tooth should be replanted, the procedure of cleaning the dirty knocked-out tooth and the way of transport of the avulsed tooth before professional treatment were answered correctly by 17.87%, 40.85% and 35.32% of participants, respectively (Table 1). With respect to the question about where to treat the knocked-out tooth, 67.66% of participants answered correctly. In the current study, the observed frequency of incorrect and correct answers was tested (Table 2). It was determined that there is a statistically significant difference (*p* < 0.05) between the observed and expected frequency.

Only 19 (8.08%) participants gave correct answers to all four knowledge-based questions. No statistically significant dependency of the number of correct answers to knowledge-based questions was found when analyzing answers about the nature of tooth injuries (*p* = 0.17), knocked-out teeth (*p* = 0.92) and tooth replantation (*p* = 0.19). Statistically significant dependency of the number of correct answers was determined among students who knew that the knocked-out tooth should be placed back into the socket (*p* = 0.01) as well those students who knew what should be done if the tooth is knocked-out and falls on the ground (*p* = 0.01)). There was a statistically significant difference among students who had been informed about the management of a knocked-out tooth (*p* = 0.01). Of all the students who had been informed, 33.33% of students did provide correct answers to all four questions about the management of a knocked-out tooth. The “I do not know” answer was chosen by 18.30% of participants whenever it was offered while the majority (89.78%) were not aware of the fact that the procedure they chose would be inappropriate.

## 4. Discussion

The current study has shown that most participants are not familiar with tooth injuries that are in accordance with the existing data from similar studies conducted in Turkey, Czech Republic, India and Brazil [16,23,24,25]. However, the majority of students felt that they knew what a knocked-out tooth and tooth replantation were and that the avulsed tooth should be put back into the socket, which is similar to the study conducted with school teachers within an urban and rural area in India [16]. The literature review has shown that respondents are often not aware that the tooth can be saved and that they would rather discard the avulsed tooth [26,27,28,29]. Only 45.53% of participants felt that they knew what should be done if the knocked-out tooth falls on the ground. This finding is similar to those observed in studies conducted in Turkey, Czech Republic, Brazil and India [16,18,23,24,25]. The information that is alarming is that the majority of respondents did not know that the avulsed tooth should be found, picked up by the crown without touching the root, rinsed briefly and put in an appropriate medium for transport, similarly to Andersson et al. [15] and Chanchala et al. [30], thus preventing a dry period longer than 5 min [31]. After that and without delay both the tooth and the child should be transported to the nearest dentist. Only 67.66% of participants were aware that professional help in case of emergency related to tooth injury should be found at the dentist nearby or in a general hospital. Additionally, the need for education on dental first aid is emphasized by the finding that 36.60% of participants would not use a wet medium for transport.

As far as the time of replantation is concerned, majority of students did not know the time span within which a tooth should be replanted. Teeth that had been replanted within 30 min of the accident remained in alveolus without resorption in 90% of cases [32]. Only 17.87% of students knew that the avulsed tooth should be put back in the socket within 30 min, which is a better result compared to similar studies in Brazil and India [16,17,18]. However, that is not satisfying, especially with regard to the fact that students participating in this study claimed more often (22.13%) that they had received information on management of a knocked-out tooth.

The period of dryness, prior to the placing of tooth in either the tooth socket or a medium for transport, is related to the chances for successful tooth replantation. The prevention of drying is of utmost importance, because it causes loss of normal physiologic metabolism and morphology of the periodontal ligament cells [33]. It is important to remember that the critical limit for dry time is 15 min [34]. Within that period at the latest, the immediate replantation or tooth storage in a wet medium for transport should be performed. There was a high percentage of normal periodontal healing in cases of short periods of dry storage that are less than 15 min [35]. The use of serial analysis of various dry times and wet times indicated that a dry storage time of 5 min or less has limited potential for early onset of resorption, but that a marked effect is seen after 15 min [34]. An additional 10 min of dryness will increase the probability of resorption by 29% [31].

The avulsed tooth should be placed in an appropriate wet medium for transport without delay if it will not be replanted at the site of the accident. The IADT guidelines recommend immediate replantation if possible, by encouraging the patient/guardian to replant the tooth. Then, the child should bite on a handkerchief to hold it in position and be taken to the nearest dentist [15]. Usually non-professionals are too scared to replant the tooth, mostly because they lack knowledge and training [19,20,21]. Additional education can encourage respondents to immediately replant the avulsed tooth. However, some other factors are present that prevent non-professionals from replanting the tooth at the site, such as some legal implications [19,20,21]. It is not probable that respondents will change their mind after being educated about replanting a tooth if they are afraid of being sued for replanting it incorrectly [20]. Clarification is required regarding issues of responsibility and acceptable levels of competence for professionals other than dentists, who may be expected to provide emergency care during the critical moments following traumatic dental injuries such as avulsion [21]. One of the arguments for choosing a transport medium instead of immediate transplantation is the possibility that the tooth will not be immobilized well in the mouth either by the patient or due to incorrect instructions from a non-professional person. The safest way to prevent either the mechanical damage at the root area, or swallowing or annihilating the tooth by accident, is to transport it out of the mouth into a suitable storage medium. Another argument for wet transport medium as the first and more appropriate choice is progress made in successful replantation of an avulsed tooth. A case of successful replantation of a tooth has been recorded even though it was kept in a plastic envelope under dry conditions for 30 h after avulsion [36], which is quite promising.

The current study has confirmed a low level of respondents’ awareness regarding the appropriate medium for tooth transport. Only 35.31% of participants would use saline water, milk, or saliva, which would create better chances for successful replantation similarly to the Bahammam (31%) [37] and Ulusoy et al. [38] studies. The 25.11% of students were aware that they did not know what an appropriate medium was. Physiological saline and cell culture media in specialized transport containers are presently considered to be impractical as they are not generally available at the accident sites where injury is likely to occur [33,39]. For that reason, it is good to be aware that avulsed teeth can be stored in a physiologic storage medium that is available near the site of an accident, such as milk or patient saliva [39]. Refrigerated milk is preferable to room temperature milk to maintain periodontal cell viability [39,40]. Regarding the temperature of media, it has been found that storage of exarticulated teeth in a physiologic medium on ice for up to one hour provides better conditions than storage for the same time and in the same medium at room temperature [41]. Reduced fat milk should be used instead of whole milk since there is evidence that milk with a lower fat content may be more appropriate for maintaining cell viability than milk with higher fat content [41,42]. Long shelf-life milk can be recommended as a suitable transport medium for avulsed teeth and it can be available in schools, gyms and outdoor athletic fields, where tooth avulsions are most likely to occur [43]. The advantage of milk over patient saliva is supported by findings that microorganisms from saliva may affect the survival of the periodontal ligament cells [44,45].

Only 40.85% of participants would use the appropriate medium for cleaning, which is less than findings within similar studies in India and Brazil [16,17,18]. Majority of students in the current study do not know what medium to use to clean the avulsed tooth if it has fallen on the ground. One of the worst scenarios was chosen by 2.98% of students. They would choose to brush both the crown and root of the avulsed tooth. The tooth should not be brushed. Cleaning the root surface would be detrimental to periodontal healing because of the mechanical damage to the periodontal ligament cells [39]. Regarding the cleaning medium, 50.64% of participants do not know that if the tooth falls on the ground it should be gently taken by the crown and washed for a period not longer than 10 s [35]. Special attention should be paid not to damage periodontal ligament cells of the root during washing [46]. A suitable cleaning medium should be the same as the medium that will be used for transport. If there is no suitable medium near the accident site, tap water can be used but the tooth should be washed briefly. Since there is no suitable medium for transport near the place of the accident, after washing it with tap water immediate replantation of the tooth to the alveolar socket should be performed as it is recommended by IADT guidelines [15].

This cross-sectional study was conducted on a sample of students from four out of six Faculty of Educations in the Republic of Croatia, which limits the possibility to generalize the findings to the whole Croatian student population. We consider this sample representative enough to emphasize the fact clearly revealed from the survey; students lack education in dental injuries and serious actions need to be undertaken to change attitudes and procedures which would usually be done by majority of students. Timely and adequate emergency procedures for traumatic injuries of orofacial structures are of crucial importance for optimal therapy success, while untreated traumas can have severe consequences [30,47,48,49,50].

## 5. Conclusions

The current study has confirmed that the participants’ responses are not random, but result from either a lack of knowledge or wrong intuition about the correct answer. Statistically significant dependency is found between the number of correct answers and questions referring to the awareness of the nature of a knocked-out tooth, to the question whether the avulsed tooth should be placed back into the socket and the previously received information on avulsed tooth management. Only 8.08% of students provided correct answers to all four questions related to pre-hospital emergency management of tooth avulsion. Some participants were aware that they did not know this and chose this answer when it was offered. The 89.78% of participants were not aware that they did not know how to provide proper dental first aid to a child with an avulsed tooth.

The knowledge evaluation regarding tooth avulsion and emergency response emphasizes the need for additional education on dental emergencies among students of the Faculty of Education. Participants are future teachers in primary schools and are expected to witness these types of injuries among children. Providing professional information on dental first aid is to become an urgent priority, aimed to improve knowledge of the emergency management of tooth avulsion. Better official communication between dental professionals and non-professionals involved in some type of childcare profession is needed, to ensure appropriate dental first aid response of non-professional persons to avulsed tooth dental emergencies.

The IADT guidelines of the pre-hospital emergency management of tooth avulsion should become an obligatory part of the education for students of the Faculty of Education. Every future childcare worker should know how to save the avulsed tooth until visiting the nearest dentist. More than three quarters of participants did not receive any information about tooth avulsion until that point, indicating the need for a successful type of education that can be applied today. Regarding that, educational campaigns dedicated to this topic are recommended and leaflets should be shared among students. In the long term, the curriculum of students of education should include the IADT guidelines as an obligatory part of the programs.

## Figures and Tables

**Table 1:** Students’ responses to the applied questionnaire.

Number of Question	Questions	Frequency N (%)
Q1	Do you know what tooth injuries are?	
a	Yes	95 (40.43)
b	No	140 (59.57)
Q2	Do you know what a knocked-out tooth is?	
a	Yes	196 (83.40)
b	No	39 (16.60)
Q3	Do you know what tooth replantation is?	
a	Yes	134 (57.02)
b	No	101 (42.98)
Q4	If the tooth is knocked-out and falls on the ground, do you know what should be done?	
a	Yes	107 (45.53)
b	No	128 (54.47)
Q5	Should the knocked-out tooth be placed back into the socket?	
a	Yes	147 (62.55)
b	No	88 (37.45)
Q6	How immediately the tooth replantation should be performed after the tooth comes out of the socket?	
a	5 min *	9 (3.83)
b	30 min *	33 (14.04)
c	1 h	25 (10.64)
d	6 h	7 (2.98)
e	24 h	20 (8.51)
f	72 h	1 (0.43)
g	I do not know	140 (59.57)
Q7	If the tooth falls on the ground and gets dirty, what should you do?	
a	Brush crown and root	7 (2.98)
b	Wash with tap water *	20 (8.51)
c	Wash with milk *	30 (12.77)
d	Wash with saline *	46 (19.57)
e	Do not wash	13 (5.53)
f	I do not know	119 (50.64)
Q8	First place to seek for replantation treatment?	
a	First aid ambulance	67 (28.51)
b	General hospital *	3 (1.28)
c	Dentist nearby *	156 (66.38)
d	Medical doctor	6 (2.55)
e	Medical College	0 (0)
f	Dental College	1 (0.43)
g	Others	2 (0.85)
Q9	Transport media?	
a	Tissue paper	49 (20.85)
b	Toilet paper	2 (0.85)
c	Cotton rolls	3 (1.28)
d	Pocket	0 (0)
e	Poly bags	32 (13.62)
f	Tap water	5 (2.13)
g	Saline water *	26 (11.06)
h	Milk *	31 (13.19)
i	Saliva *	26 (11.06)
j	Others	2 (0.85)
k	I do not know	59 (25.11)
Q10	Have you ever received any kind of information on management of knocked-out tooth?	
a	Yes	52 (22.13)
b	No	183 (77.87)

* correct answer.

**Table 2:** Distribution of incorrect and correct response frequency on knowledge-based questions.

Questions	Incorrect Response	Correct Response	*p*-Value	“I Do Not Know”
Q6	53	42	0.02 *	140
Q7	20	96	0.01 *	119
Q8	76	159	0.01 *	
Q9	93	83	0.01 *	59

* significant difference (Chi-square, *p* < 0.05).

## References

[1] Petersson E.E., Andersson L., Sorensen S. (1997). Traumatic oral vs. non-oral injuries. Swed. Dent. J..

[2] Glendor U., Halling A., Andersson L., Eilert-Peterson E. (1996). Incidence of traumatic tooth injuries in children and adolescents in the county of Vastmanland, Sweden. Swed. Dent. J..

[3] Glendor U. (2009). Has the education of professional caregivers and lay people in dental trauma care failed?. Dent. Traumatol..

[4] Andreasen J.O., Andreasen F.M., Skeie A., Hjorting-Hansen E., Schwartz O. (2002). Effect of treatment delay upon pulp and periodontal healing of traumatic dental injuries—A review article. Dent. Traumatol..

[5] Andersson L., Bodin I., Sorensen S. (1989). Progression of root resorption following replantation of human teeth after extended extra-oral storage. Endod. Dent. Traumatol..

[6] Andersson L., Bodin I. (1990). Avulsed human teeth replanted within 15 minutes-a long-term clinical follow-up study. Endod. Dent. Traumatol..

[7] Al Jundi S.H. (2002). Dental emergencies presenting to a dental teaching hospital due to complications from traumatic dental injuries. Dent. Traumatol..

[8] Al Jundi S.H. (2004). Type of treatment, prognosis, and estimation of time spent to manage dental trauma in late presentation cases at a dental teaching hospital: A longitudinal and retrospective study. Dent. Traumatol..

[9] Andersson L., Malmgren B. (1999). The problem of dentoalveolar ankylosis and subsequent replacement resorption in the growing patient. Aust. Endod. J..

[10] Kargul B., Welbury R. (2009). An audit of the time to initial treatment in avulsion injuries. Dent. Traumatol..

[11] Cohenca N., Stabholz A. (2007). Decoronation—A conservative method to treat ankylosed teeth for preservation of alveolar ridge prior to permanent prosthetic reconstruction: Literature review and case presentation. Dent. Traumatol..

[12] Glendor U., Jonsson D., Halling A., Lindqvist K. (2001). Direct and indirect costs of dental trauma in Sweden: A 2-year prospective study of children and adolescents. Community Dent. Oral Epidemiol..

[13] Andreasen J.O. (1970). Etiology and pathogenesis of traumatic dental injuries. A clinical study of 1298 cases. Scand. J. Dent. Res..

[14] Bastone E.B., Freer T.J., McNamara J.R. (2000). Epidemiology of dental trauma: A review of the literature. Aust. Dent. J..

[15] Andersson L., Andreasen J.O., Day P., Heithersay G., Trope M., Diangelis A.J., Kenny D.J., Sigurdsson A., Bourguignon C., Flores M.T. (2012). International Association of Dental Traumatology guidelines for the management of traumatic dental injuries: 2. Avulsion of permanent teeth. Dent. Traumatol..

[16] Kaur M., Gupta K., Goyal R., Chaudhary N. (2014). Knowledge and Attitude of School Teachers towards Tooth Avulsion in Rural and Urban Areas. Int. J. Sci. Study.

[17] Prasanna S., Giriraju A., Narayan N.L. (2011). Knowledge and Attitude of Primary School Teachers toward Tooth Avulsion and Dental First Aid in Davangere City: A Cross-sectional Survey. Int. J. Clin. Pediatric Dent..

[18] Panzarini S.R., Pedrini D., Brandini D.A., Poi W.R., Santos M.F., Correa J.P.T., Silva F.F. (2005). Physical education undergraduates and dental trauma knowledge. Dent. Traumatol..

[19] Blakytny C., Surbuts C., Thomas A., Hunter M.L. (2001). Avulsed permanent incisors: Knowledge and attitudes of primary school teachers with regard to emergency management. Int. J. Pediatr. Dent..

[20] Hamilton F.A., Hill F.J., Mackie I.C. (1997). Investigation of lay knowledge of the management of avulsed permanent incisors. Endod. Dent. Traumatol..

[21] Addo M.E., Parekh S., Moles D.R., Roberts G.J. (2007). Knowledge of dental trauma first aid (DTFA): The example of avulsed incisors in casualty departments and schools in London. Br. Dent. J..

[22] Ivancic Jokic N., Bakarcic D., Grzic R., Majstorovic M., Sostarek M. (2017). What general medicine students of University of Rijeka know about dental avulsion?. Eur. J. Dent. Educ..

[23] Çaglar E., Ferreira L.P., Kargul B. (2005). Dental trauma management knowledge among a group of teachers in two south European cities. Dental. Traumatol..

[24] Tzigkounakis V., Merglova V. (2008). Attitude of Pilsen primary school teachers in dental trauma. Dent. Traumatol..

[25] Pacheco L.F., Filho P.F.G., Letra A., Menezes R., Villoria G.E.M., Ferreira S.M. (2003). Evaluation of the knowledge of the treatment of avulsions in elementary school teachers in Rio de Janeiro, Brazil. Dent. Traumatol..

[26] Gupta G., Gupta N., Chopra R., Sachde V. (2016). Awareness towards management and prevention of dental injuries among sports instructors in Delhi, India. J. Dent. Specialities.

[27] Yunus G.Y., Nalwar A., Divya Priya G.K., Veeresh D.J. (2015). Influence of educational intervention on knowledge and attitude toward emergency management of traumatic dental injuries among nursing students in Davangere, India: Pre- and post-design. J. Indian Assoc. Public Health Dent..

[28] Nikam A.P., Kathariya M.D., Chopra K., Gupta A., Kathariya R. (2014). Knowledge and Attitude of Parents/Caretakers toward Management of Avulsed Tooth in Maharashtrian Population: A Questionnaire Method. J. Int. Oral Health.

[29] Kaur H., Kaur S., Kaur H. (2012). Prehospital emergency management of avulsed permanent teeth: Knowledge and attitude of school teachers. Indian J. Dent. Res..

[30] Chanchala H.P., Shanbhog R., Ravi M.D., Raju V. (2016). Pediatricians’ perspectives on dental trauma management: A cross-sectional survey. J. Indian Assoc. Public. Health Dent..

[31] Kinirons M.J., Gregg T.A., Welbury R.R., Cole B.O. (2000). Variations in the presenting and treatment features in reimplanted permanent incisors in children and their effect on the prevalence of root resorption. Br. Dent. J..

[32] Andreasen J.O., Hjorting-Hansen E. (1966). Replantation of teeth. I. Radiographic and clinical study of 110 human teeth replanted after accidental loss. Acta Odontol. Scand..

[33] Trope M. (2002). Clinical management of the avulsed tooth: Present strategies and future directions. Dent. Traumatol..

[34] Donaldson M., Kinirons M.J. (2001). Factors affecting the time of onset of resorption in avulsed and replanted incisor teeth in children. Dent. Traumatol..

[35] Chappuis V., von Arx T. (2005). Replantation of 45 avulsed permanent teeth: A 1-year follow-up study. Dent. Traumatol..

[36] Rahbar M., Hassani-Dehkharghani A. (2016). Replantation of an Avulsed Tooth 30 Hours after Traumatic Injury. J. Int. Oral Health.

[37] Bahammam L.A. (2018). Knowledge and attitude of emergency physician about the emergency management of tooth avulsion. BMC Oral Health.

[38] Ulusoy A.T., Onder H., Cetin B., Kaya S. (2012). Knowledge of medical hospital emergency pshysicians about the first-aid management of traumatic tooth avulsion. Int. J. Paediatric Dent..

[39] Barrett E.J., Kenny D.J. (1997). Avulsed permanent teeth: A review of the literature and treatment guidelines. Endod. Dent. Traumatol..

[40] Lekic P., Kenny D., Moe H.K., Barretti E., McCulloch C.A. (1996). Relationship of clonogenic capacity to plating efficiency and vital dye staining of human periodontal ligament cells: Implications for tooth replantation. J. Periodontal Res..

[41] Sigalas E., Regan J.D., Kramer P.R., Witherspoon D.E., Opperman L.A. (2004). Survival of human periodontal ligament cells in media proposed for transport of avulsed teeth. Dent. Traumatol..

[42] Harkacz O.M., Carnes D.L., Walker W.A. (1997). Determination of periodontal ligament cell viability in the oral rehydration fluid Gatorade and milks of varying fat content. J. Endod..

[43] Marino T.G., West L.A., Liewehr F.R., Mailhot J.M., Buxton T.B., Runner R.R., McPherson J.C. (2000). Determination of periodontal ligament cell viability in long shelf-life milk. J. Endod..

[44] Lindskog S., Blomlöf L. (1982). Influence of osmolality and composition of some storage media on human periodontal ligament cells. Acta Odontol. Scand..

[45] Poi W.R., Sonoda C.K., Martins C.M., Melo M.E., Pellizzer E.P., de Mendonca M.R., Panzarini S.R. (2013). Storage media for avulsed teeth: A literature review. Braz. Dent. J..

[46] Chamorro M.M., Regan J.D., Opperman L.A., Kramer P.R. (2008). Effect of storage media on human periodontal ligament cell apoptosis. Dent. Traumatol..

[47] Andreasen J.O., Andreasen F.M., Andersson L. (2018). Textbook and Color Atlas of Traumatic Injuries to the Teeth.

[48] Zampogna S., De Filippo S., Talarico V., Aloe M., Severini N., Pizzi S., De Filippo M., Polimeni A. (2014). First aid in dental trauma in pediatric age. Ital. J. Pediatr..

[49] Lin S., Levin L., Emodi O., Fuss Z., Peled M. (2006). Physician and emergency medical technicians’ knowledge and experience regarding dental trauma. Dent. Traumatol..

[50] Da Silva A.C., Passeri L.A., Mazzonetto R., De Moraes M., Moreira R.W.F. (2004). Incidence of dental trauma associated with facial trauma in Brazil: A 1-year evaluation. Dent. Traumatol..

